# Abscisic Acid Mediates Grafting-Induced Cold Tolerance of Watermelon *via* Interaction With Melatonin and Methyl Jasmonate

**DOI:** 10.3389/fpls.2021.785317

**Published:** 2021-12-17

**Authors:** Yanliang Guo, Jingyi Yan, Zhuangzhuang Su, Jingjing Chang, Jianqiang Yang, Chunhua Wei, Yong Zhang, Jianxiang Ma, Xian Zhang, Hao Li

**Affiliations:** State Key Laboratory of Crop Stress Biology for Arid Areas, College of Horticulture, Northwest A&F University, Yangling, China

**Keywords:** abscisic acid, antioxidant potential, cold, grafting, melatonin, methyl jasmonate, watermelon

## Abstract

Grafting is widely used to increase plant defense responses to various stresses. Grafting-induced cold tolerance is associated with the increase of the antioxidant potential of plants; however, the underlying mechanisms remain unclear. Here, we found that pumpkin rootstocks promote antioxidant enzyme activities and alleviate cold-induced oxidative damage, accompanied by increased abscisic acid (ABA), melatonin, and methyl jasmonate (MeJA) levels in leaves. Increased ABA accumulation in leaves was attributed partly to the increased ABA levels in rootstocks. ABA induced antioxidant enzymes activities and the accumulation of melatonin and MeJA, while inhibition of ABA synthesis blocked the rootstock-induced antioxidant activity and the accumulation of melatonin and MeJA under cold stress. Melatonin and MeJA application also enhanced ABA accumulation in leaves after cold exposure, whereas inhibition of melatonin or MeJA synthesis attenuated the rootstock-induced increase of ABA. Moreover, melatonin and MeJA application alleviated cold-induced oxidative stress, but inhibition of melatonin or MeJA synthesis lowered the rootstock- or ABA-induced antioxidant potential and tolerance to cold. These findings indicate that ABA plays an important role in the grafting-induced cold tolerance by promoting the accumulation of melatonin and MeJA, which in turn, promote ABA accumulation, forming a positive feedback loop.

## Introduction

As sessile organisms, plants frequently suffer a variety of environmental stresses throughout their life. Cold stress, one of the most severe abiotic stresses, adversely affects plant growth and development. When the ambient temperature drops, air temperature declines faster than soil temperature and thus the above-ground parts of plants are more vulnerable to cold stress. Cold stress directly impairs multiple physiological processes and indirectly initiates oxidative damage through excessive production of harmful reactive oxygen species (ROS) such as O_2_·^−^ and H_2_O_2_ (Chinnusamy et al., 2007). Excess ROS can cause oxidative stress leading to lipid peroxidation damage, DNA strand breakage, protein denaturation, enzyme deactivation, and, finally, cell death (Bose et al., 2014). To ensure their adaptation and fitness under cold stress, plants have evolved sophisticated defense mechanisms.

In response to diverse environmental stresses, roots and shoots are nonindependent but communicate to optimize the plants’ adaptation and resistance to stress. Root-to-shoot communication requires various long-distance signals such as RNAs, peptides, phytohormones, ROS, Ca^2+^, and electrical signals (Wang et al., 2019). For instance, in response to dehydration stress, root-derived signals such as peptides, hydraulic signals, ROS, and Ca^2+^ move to the leaves to promote stomatal closure and thereby reduce water loss (Yoshida et al., 2021).

Grafting is widely used to increase plant defense responses to various biotic or abiotic stresses and improve the production of various horticultural crops. In addition, grafting is also a crucial research tool to investigate the signal transduction mechanisms involved in root-to-shoot communication. By using grafting, Wang et al. (2019) found that root-knot nematode attacks induce the transmission of electrical and ROS signals from roots to leaves, resulting in increased JA accumulation and increased resistance.

Watermelon (*Citrullus lanatus*) is one of the most valuable vegetable crops worldwide. In 2018, it was cultivated on approximately 3.24 million hectares and reached a production of 103.93 million tons.^1^ As a thermophilic crop, watermelon is highly sensitive to cold stress (Rivero et al., 2001), which significantly limits the productivity of watermelon in winter or early spring. Our recent study has revealed that grafting onto tolerant rootstocks induces watermelon tolerance to cold stress, accompanied by increased melatonin and methyl jasmonate (MeJA) levels (Li et al., 2021a). However, the mechanism by which tolerant rootstocks induce melatonin and MeJA accumulation of shoots in response to cold is still unclear.

The phytohormone abscisic acid (ABA) is a central regulator of plant defense against abiotic stresses (Danquah et al., 2014). In plants, pyruvate and glyceraldehydes-3-phosphate are catalyzed to form the early C_5_ precursor, which were catalyzed into xanthoxin (the C_15_ precursor of ABA) by a series of enzymes, such as 9-cis-epoxycarotenoid dioxygenase (NCED; Wasilewska et al., 2008). Xanthoxin is converted to ABA by multiple ways, such as *via* abscisic alcohol, abscisic aldehyde, or xanthoxic acid (Danquah et al., 2014). Several studies have reported the interactions between ABA and melatonin or MeJA during plant response to abiotic stresses (Anderson et al., 2004; Adie et al., 2007; Li et al., 2015; Zhao et al., 2017). Caffeic acid *O*-methyltransferase (COMT) and allene oxide cyclase (AOC) are key enzymes for melatonin and MeJA biosynthesis, respectively, in plants (Pratiwi et al., 2017; Li et al., 2021a). The results presented by Hu et al. indicate that the ABA-induced increase in antioxidant capacity and salt tolerance of tomato plants is associated with the increased accumulation of melatonin (Hu et al., 2021). Wang et al. have demonstrated that ABA mediates red light-induced biosynthesis of jasmonic acids (JAs), which act downstream of ABA, to induce cold tolerance in tomato (Wang et al., 2016).

ABA can be generated in both shoots and roots (Schachtman and Goodger, 2008). Root-originated ABA acts as a long-distance signal that can move to shoots, where it elicits defense responses to various abiotic stresses (Schachtman and Goodger, 2008; Ntatsi et al., 2013; Li et al., 2018). In grafted cucumber plants, luffa rootstock-sourced ABA induces shoot tolerance against heat by regulating the expression of *HEAT SHOCK PROTEIN 70* and csa-miR159b (Li et al., 2014a, 2016). These findings support the assumption that ABA also plays an essential role in the rootstock-enhanced cold tolerance of shoots. To examine this hypothesis, we studied the role of ABA and its crosstalk with melatonin and MeJA in rootstock-enhanced shoot tolerance to cold stress. Our results revealed that ABA functions together with melatonin and MeJA to stimulate the antioxidant potential and cold tolerance in grafted watermelon seedlings. These findings provide new insights into the mechanisms underlying grafting-enhanced cold tolerance.

## Materials and Methods

### Plant Materials

In this study, watermelon [*Citrullus lanatus* (Thunb.) Matsum. & Nakai] cultivar Nongkeda No. 5 and pumpkin (*Cucurbita moschata*) cultivar Weizhen No. 1 were used. Watermelon and pumpkin seeds for rootstocks were directly planted in trays filled with commercial peat-based compost. Watermelon seeds for scion were sown with a seven-day delay. Once the scion cotyledons expanded, top insertion grafts were prepared (Davis et al., 2008). The grafted plants with watermelon or pumpkin rootstocks were designated as *Cl* or *Cm*, respectively. The plants were cultured under the following conditions: 25°C/18°C (day/night) temperature, 12 h/12 h (light/dark) photoperiod, 400 μmol m^−2^ s^−1^ photosynthetic photon flux density (PPFD), 65–75% relative humidity. The grafted plants were watered every 2 days and supplied with half-strength Hoagland’s nutrient solution every 6 days.

### Experimental Design

Four-leaf stage *Cl* and *Cm* seedlings were transferred into growth chambers at 25°C for the control treatment or 4°C for the cold treatment. At 12 h after exposure to 4°C, the leaf and root samples and xylem sap exudates were collected for hormone quantification and gene expression assays. At 36 h, the redox homeostasis and cold tolerance of plants were analyzed.

To investigate the effects of exogenous ABA, melatonin, or MeJA on plant tolerance to cold-induced oxidative stress, each *Cl* seedling was sprayed with 20 ml ABA (100 μm; Fu et al., 2017), melatonin (150 μm; Li et al., 2021a), MeJA (200 μm; Li et al., 2021a). For preparation of these solutions, the ABA (CAS: 14375-45-2, 98%), melatonin (CAS: 73-31-4, ≥98%), or MeJA (CAS: 39924-52-2, ≥98%), purchased from Sigma-Aldrich (St. Louis, MO, United States), was dissolved in absolute ethanol, followed by a 1/10,000 (v/v) dilution in distilled water. The same proportion of diluted absolute ethanol in distilled water was used as the control. After 12 h, the seedlings were transferred to 4°C. To inhibit the biosynthesis of melatonin or MeJA, the *Cl* plants were sprayed with 100 μm p-chlorophenyl alanine (CPA, an inhibitor of melatonin biosynthesis, CAS: 7424-00-2, Sigma-Aldrich, ≥98.5%; Park, 2011; Wen et al., 2016) or 5 mm diethyldithiocarbamic acid (DIECA, an inhibitor of JA synthesis, CAS: 1518-58-7, Sigma-Aldrich, 97%; Hu et al., 2003), respectively. CPA and DIECA solutions were prepared as ABA. After 8 h, the plants were treated with ABA. Twelve hours later, the seedlings were transferred to 4°C. The leaf samples were taken at 12 h after cold treatment for hormone quantification and gene expression assays and at 36 h for cold tolerance analysis.

To determine the involvement of ABA, melatonin, and MeJA in pumpkin rootstock-alleviated, cold-induced oxidative stress, *Cm* plants were sprayed with 50 μm fluridone (an ABA synthesis inhibitor; CAS: 59756-60-4, Sigma-Aldrich, ≥98%; Fu et al., 2017), CPA, or DIECA, respectively. Fluridone solution was prepared as ABA. In addition to fluridone spraying, the roots of each plant were watered with 50 ml of 25 μm fluridone. After 8 h, the plants were transferred to 4°C. The leaf samples were taken at 12 h after cold treatment for hormone quantification and gene expression assays and at 36 h for cold tolerance analysis.

### Analysis of the Net Photosynthetic Rate (Pn) and Chlorophyll Fluorescence

The Pn was measured on the third leaf from the bottom of seedlings using a portable photosynthesis system (LI-6400, Li-Cor, Lincoln, NE, United States) under the following conditions: 25°C temperature, 400 μmol m^−2^ s^−1^ PPFD, and 380 μmol mol^−1^ CO_2_. The maximal quantum yield of photosystem (PS) II (*Fv/Fm*) and the quantum yield of PSII [Y(II)] were determined by using a pulse-amplitude modulated chlorophyll fluorometer (imaging-PAM, Heinz Walz GmbH, Effeltrich, Germany) after plants were kept in the dark for 30 min.

### Quantification of ABA, Melatonin, and Meja and Xylem Sap Collection

ABA and MeJA were extracted following the methods described previously (Yang et al., 2001; Pan et al., 2010). In brief, 0.5 g of frozen samples was homogenized in 5 ml of 1-propanol/H_2_O/concentrated HCl (2/1/0.002, v/v/v). After overnight incubation, the extracts were mixed with 5 ml of dichloromethane. The mixture was shaken for 30 min at 4°C. After centrifugation at 15,000 *g* for 5 min at 4°C, the obtained lower phase was dried using N_2_ gas. The residue was then dissolved in methanol. The concentrations of ABA or MeJA were measured using an ELISA kit according to the manufacturer’s instructions (China Agricultural University, Beijing, China).

Melatonin was extracted using a method developed by Pape and Lüning (2006). Frozen leaf samples (0.5 g) were homogenized in 5 ml of acetone–methanol buffer (acetone/methanol/water = 89/10/1, v/v/v) on ice. The homogenate was centrifuged at 5,000 *g* for 10 min at 4°C. Then the supernatant was mixed with 0.5 ml of trichloric acid (1%). After centrifugation at 10,000 *g* for 10 min at 4°C, the melatonin level was measured using an ELISA kit (Shanghai Lanpai Biotech Co., Ltd., Shanghai, China).

For xylem sap collection, the stem was cut 5 cm above the soil surface, and the cut surface was blotted dry 3 times using sterile filter paper. The xylem sap exudates were collected using micropipette tips (Li et al., 2014a). ABA exudation rate was calculated according to the sap exudation rate and ABA content in xylem sap (Supplementary Figure S1).

### Analysis of H_2_O_2_, O_2_·^−^, Malondialdehyde, and Relative Electric Conductivity

Analysis of H_2_O_2_ was conducted as reported in Willekens et al. (1997). H_2_O_2_ was extracted from leaf samples (0.3 g) in 1 M HClO_4_ (3 ml). After centrifugation at 6,000 *g* for 5 min at 4°C, the supernatants were adjusted to pH 6.0–7.0 with KOH (4 M) and filtered using Bio-Rad AG1x8 columns (Hercules, CA, United States). After elution with double distilled water, the samples (800 μl) were mixed with 400 μl 100 mm potassium acetate buffer (pH 4.4) containing 4 mm 2,2′-azino-di (3-ethylbenzthiazoline-6-sulfonic acid), 400 μl deionized water, and 0.25 U of horseradish peroxidase. The H_2_O_2_ content was detected at OD_412_.

O_2_·^−^ analysis was performed as reported in Elstner and Heupel (1976). Leaf samples (0.5 *g*) were homogenized in 3 ml of 65 mm potassium phosphate buffer (pH 7.8). After centrifugation, the supernatant (1 ml) was mixed with 65 mm phosphate buffer (pH 7.8, 0.9 ml) and 10 mm hydroxylamine hydrochloride (0.1 ml). The mixture was incubated at 25°C for 20 min and then, they were mixed with 17 mm sulfanilamide and 7 mm a-naphthylamine. After reaction at 25°C for 20 min, the mixture was mixed with ethyl ether in the same volume. After centrifugation at 1,500 *g* for 5 min, the O_2_·^−^ production was detected at OD_530_.

Malondialdehyde (MDA), as an index of lipid peroxidative damage, was determined using 2-thiobarbituric acid following the method of Hodges et al. (1999). The relative electric conductivity (REC) was determined following the method of Zhou and Leul (1998).

### Analysis of Antioxidant Enzyme Activities

The activities of antioxidant enzymes in leaves were analyzed using spectrophotometric methods. For enzyme extraction, leaf samples (0.3 *g*) were ground in 3 ml 25 mm HEPES buffer (pH 7.8, 0.2 mm EDTA, 2 mm ascorbic acid, and 2% polyvinyl pyrrolidone) on ice. Superoxide dismutase (SOD) activity was analyzed following the method developed by Stewart and Bewley (1980) based on photochemical reduction of NBT. Peroxidase (POD) activity was measured following the method of Cakmak and Marschner (1992). Catalase (CAT) activity was measured as a decline in A240 according to the method of Patra et al. (1978). Developed by Stewart and Bewley (1980).

### Analysis of Gene Expression

Total RNA was extracted using the RNA extraction kit from Axgen (Union City, CA, United States) according to the manufacturer’s instructions. After extraction, the RNA samples were treated with gDNase to remove DNA, then reverse-transcribed (1 μg per sample) to cDNA using a ReverTra Ace qPCR RT kit (Toyobo, Osaka, Japan). Quantitative Real-Time PCR was conducted using SYBR Premix ExTaqII (2×) Kit (Takara, Tokyo, Japan) on an iCycler Iq TM Multicolor PCR Detection System (Bio-Rad, Hercules, CA, United States; Li et al., 2017). *β-ACTIN* was used as an internal control gene. Primers used for gene expression analyses are listed in Supplementary Table S1. The relative expression of genes was calculated using the 2^−ΔΔCT^ method as reported in Livak and Schmittgen (2001).

### Statistical Analysis

The experiments were laid out in a completely randomized design. All experiments were repeated three times, and each experiment contained at least 15 seedlings per treatment. Data are presented as means ± standard deviations (SD). The differences among treatments were determined *via* one-way or two-way variance (ANOVA) using SPSS package (SPSS 19.0, Chicago, IL, United States), followed by Tukey’s test at *p* < 0.05. Significant differences were indicated by different letters.

## Results

### Pumpkin Rootstocks Alleviate Cold-Induced Oxidative Damage in Watermelon Leaves

**Figure 1:**
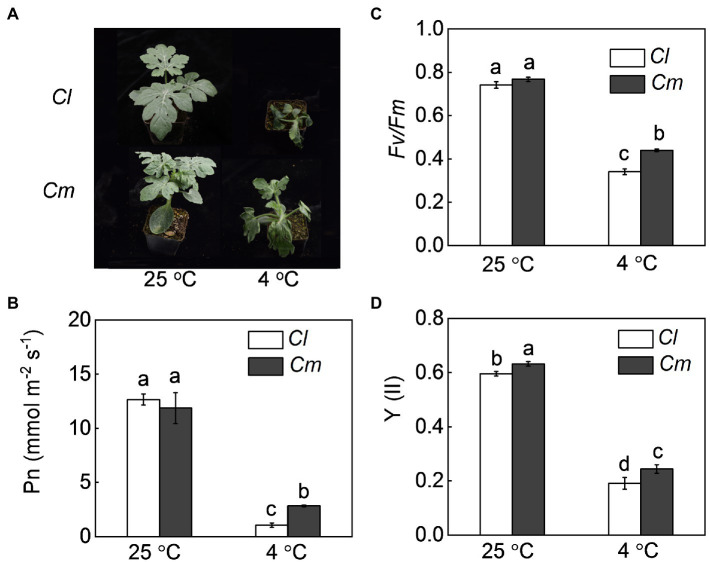
Pumpkin rootstock-alleviated cold-induced wilting and decrease of photosynthetic performance in watermelon shoots. Self-grafted (*Cl*) and pumpkin-grafted (*Cm*) watermelon plants were exposed to cold stress at 4°C. After cold treatment for 36 h, the **(A)** phenotypes, **(B)** net photosynthetic rate (Pn), **(C)** photochemical efficiency of PSII (*Fv/Fm*), and **(D)** quantum yield of PSII [Y(II)] were analyzed. Data are reported as means ± standard deviations (*n* = 6). Different letters indicated significant difference at *p* < 0.05.

We examined the changes in the plant phenotypes, Pn, *Fv/Fm*, and Y(II) of watermelon shoots grafted onto watermelon (*Cl*) or pumpkin (*Cm*). Under normal conditions, no significant differences were found in seedling growth, Pn, or *Fv/Fm* between *Cl* and *Cm* plants (Figure 1). Exposure to 4°C resulted in obvious plant wilting and significant decreases in Pn, *Fv/Fm*, and Y(II) in watermelon leaves. However, pumpkin rootstocks attenuated the cold-induced wilting and reduction in Pn, *Fv/Fm*, and Y(II). After exposure to 4°C for 36 h, Pn, *Fv/Fm*, and Y(II) in *Cm* plants were 168.3, 29.2, and 27.7% higher than in *Cl* plants, respectively.

**Figure 2:**
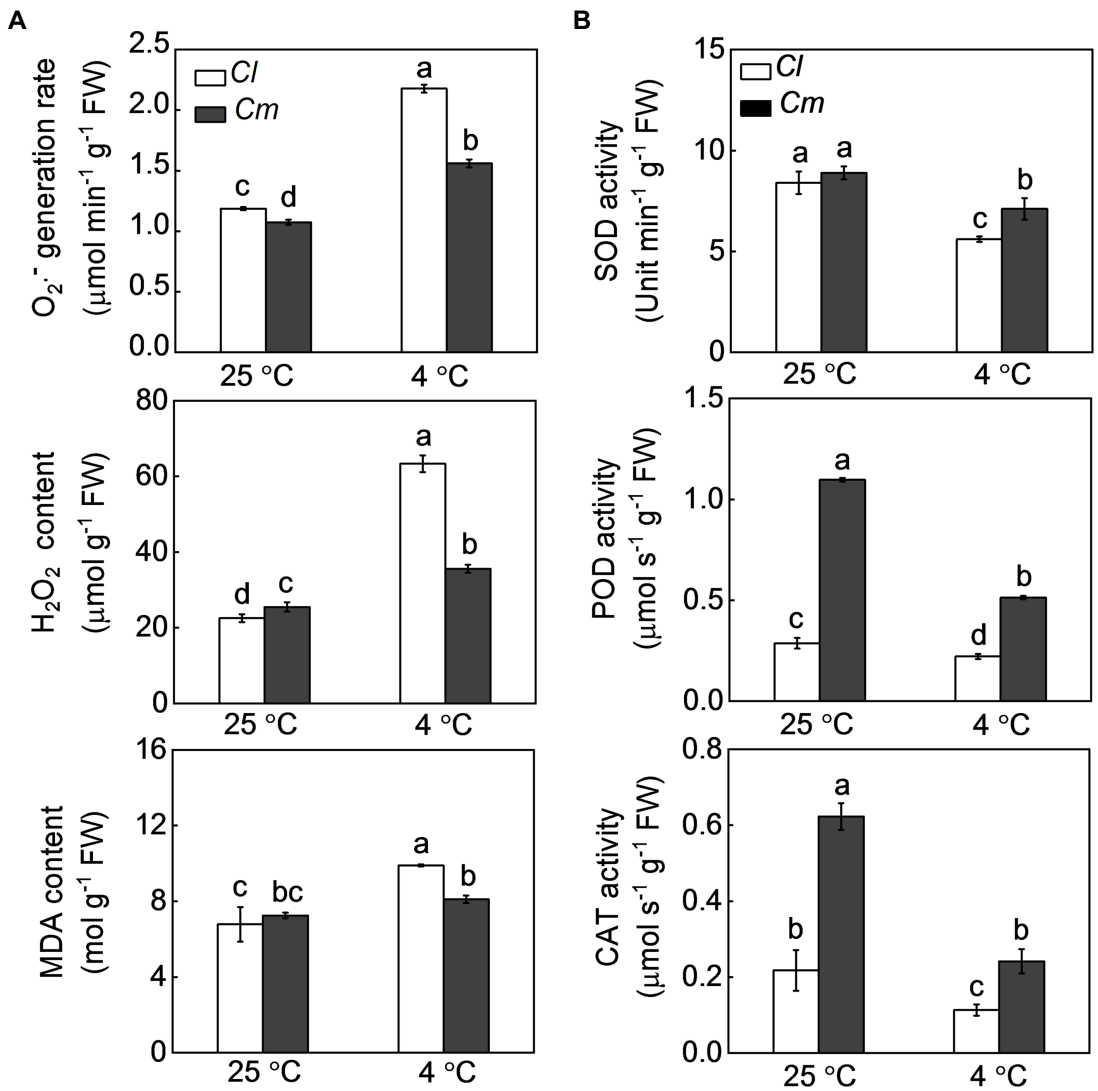
Pumpkin rootstock-alleviated cold-induced oxidative damage in watermelon shoots. The seedlings were treated according to the procedures outlined in Figure 1. **(A)**, The levels of O_2_·^−^, H_2_O_2_, and malondialdehyde (MDA). **(B)** The activities of superoxide dismutase (SOD), peroxidase (POD), and catalase (CAT). Data are reported as means ± standard deviations (*n* = 3). Different letters indicated significant difference at *p* < 0.05.

We then investigated the effects of the pumpkin rootstock on the antioxidant potential and oxidative damage in watermelon plants in response to cold. Under normal conditions, *Cl* and *Cm* plants showed similar generation rates of O_2_·^−^ and similar levels of H_2_O_2_ and MDA. Cold exposure caused a significant increase of O_2_·^−^, H_2_O_2_, and MDA levels, which was more apparent in *Cl* plants than in *Cm* plants (Figure 2A). SOD, POD, and CAT are well-studied, key antioxidant enzymes that can scavenge excess ROS. In *Cl* and *Cm* plants, SOD, POD, and CAT were significantly reduced by cold (Figure 2B). Interestingly, *Cm* plants exhibited higher activities of these three enzymes than *Cl* plants under cold stress. For instance, SOD, POD, and CAT activities in *Cm* plants were 26.8, 131.8, and 113.1% higher than in *Cl* plants after exposure to cold, respectively.

### Involvement of ABA in the Rootstock-Induced Increase of Melatonin and Meja Accumulation

**Figure 3:**
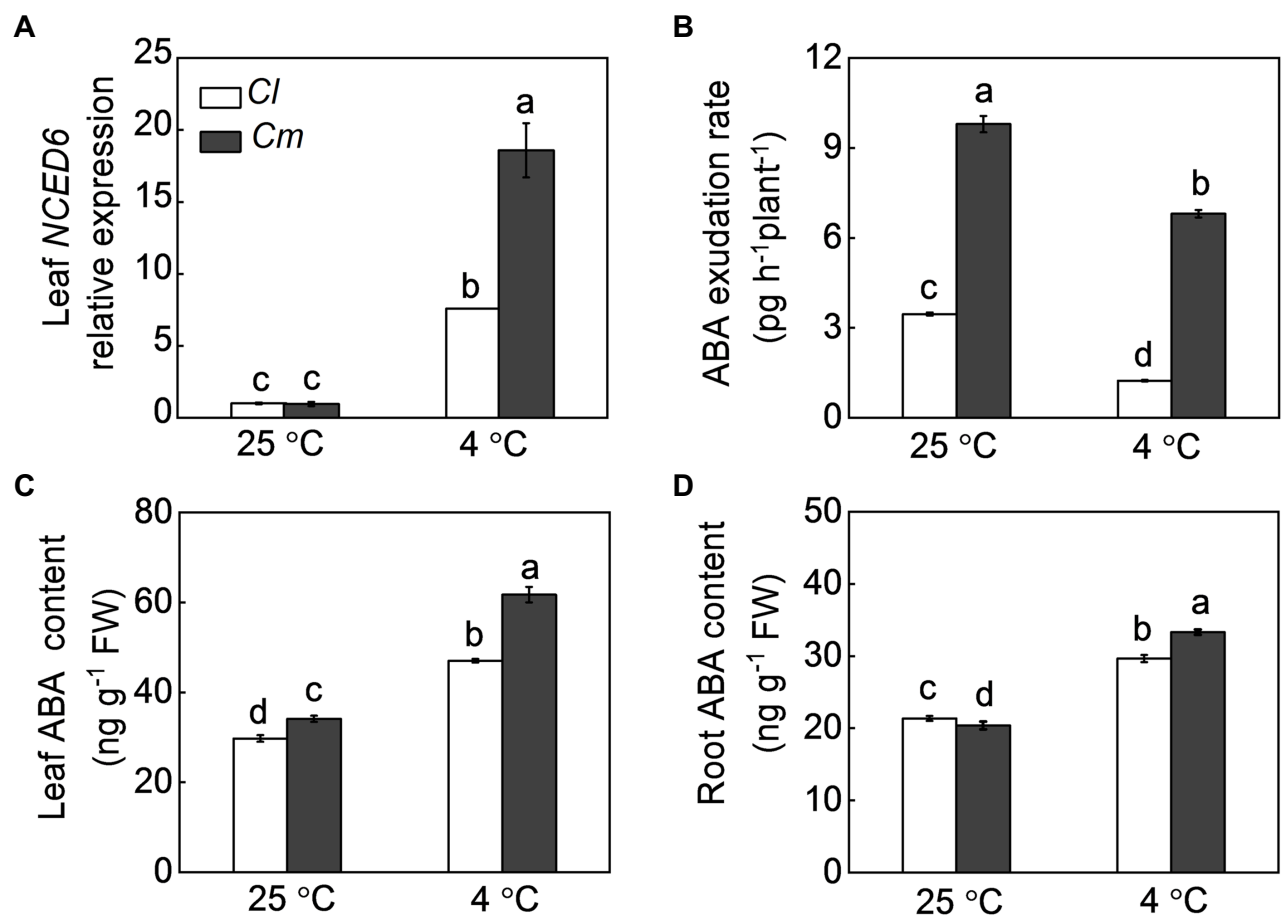
ABA accumulation and transport *via* the xylem in response to cold in grafted watermelon plants. **(A)** The transcript levels of *9-CIS-EPOXYCAROTENOID DIOXYGENASE 6* (*NCED6*) in leaves. **(B)** ABA contents in leaves. **(C)** ABA exudation rates from the xylem. **(D)** ABA contents in roots. Self-grafted (*Cl*) and pumpkin-grafted (*Cm*) watermelon plants were exposed to cold stress at 4°C. Samples of roots, leaves, and xylem sap were collected at 12 h after cold exposure. Data are reported as means ± standard deviations (*n* = 3). Different letters indicated significant difference at *p* < 0.05.

As ABA is an essential root-to-shoot signal in plant defense against various environmental stresses, the response of ABA to cold stress was analyzed in grafted plants. Under normal conditions, *Cm* plants showed higher ABA content in leaves and a higher ABA exudation rate than *Cl* plants (Figure 3). In *Cl* and *Cm* plants, cold stress increased the accumulation of ABA in roots and leaves and the transcript levels of *NCED6* in leaves but reduced the rate of ABA exudation from the xylem. Interestingly, *Cm* plants showed higher *NCED6* transcript levels in leaves, ABA accumulation in roots and leaves, and ABA exudation rates than *Cl* plants after exposure to cold.

**Figure 4:**
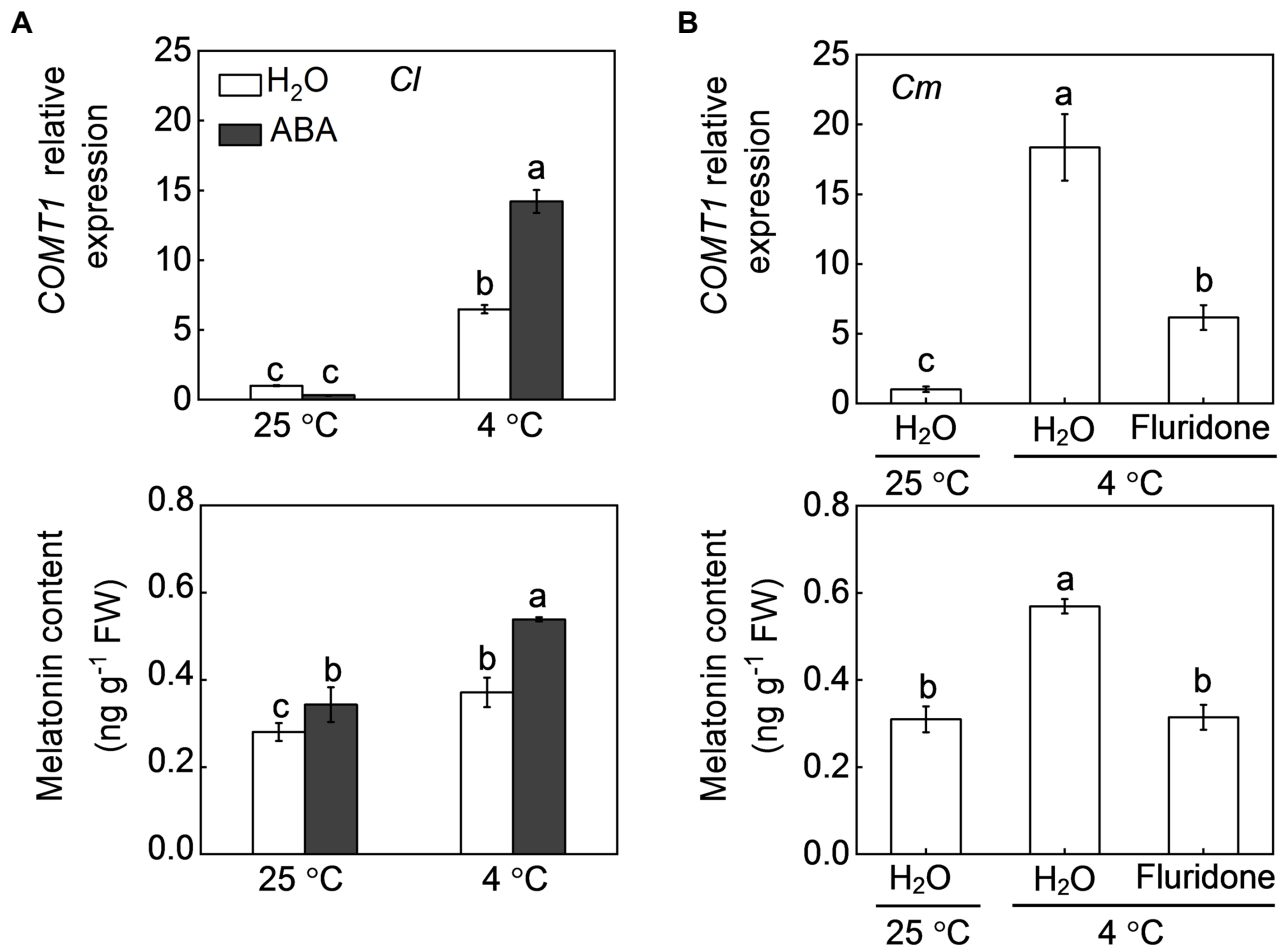
Involvement of ABA in pumpkin rootstock-mediated melatonin accumulation in watermelon leaves in response to cold. **(A)** The effects of ABA on the levels of the *CAFFEIC ACID O-METHYLTRANSFERASE 1* (*COMT1*) transcript and melatonin in leaves of self-grafted plants (*Cl*) under cold exposure. The leaves of *Cl* seedlings were sprayed with 100 μm ABA or H_2_O (Control). Twelve hours later, the seedlings were exposed to cold stress at 4°C for 12 h. **(B)** The effects of fluridone on *COMT1* transcript and melatonin levels in leaves of pumpkin-grafted plants (*Cm*) under cold exposure. The leaves of *Cm* seedlings were sprayed with 50 μm fluridone. Meanwhile, the seedlings were irrigated with 25 μm fluridone. Eight hours later, the seedlings were exposed to cold stress at 4°C for 12 h. Data are reported as means ± standard deviations (*n* = 3). Different letters in **(A)** and **(B)** indicated significant difference at *p* < 0.05.

**Figure 5:**
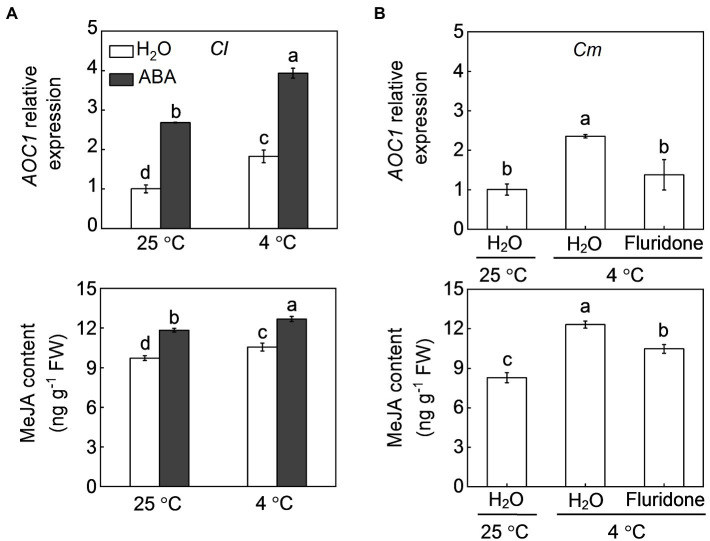
Involvement of ABA in pumpkin rootstock-induced methyl jasmonate (MeJA) accumulation in watermelon shoots under cold stress. **(A)** The effects of ABA on the transcript levels of *ALLENE OXIDE CYCLASE 1* (*AOC1*) and MeJA accumulation in leaves of self-grafted plants (*Cl*) under cold exposure. **(B)** The effects of fluridone on the transcript levels of *AOC1* and MeJA accumulation in leaves of pumpkin-grafted plants (*Cm*) under cold exposure. The seedlings were treated according to the procedures outlined in Figure 4. Data are reported as means ± standard deviations (*n* = 3). Different letters in **(A)** and **(B)** indicated significant difference at *p* < 0.05.

Pumpkin rootstocks increased melatonin contents and the transcript levels of *CAFFEIC ACID O-METHYLTRANSFERASE 1* (*COMT1*), a key gene in melatonin synthesis, in watermelon leaves under cold stress (Figure 4; Li et al., 2021a). Similar to pumpkin rootstocks, exogenous ABA at 100 μm promoted melatonin accumulation in watermelon leaves under both normal conditions and cold stress and upregulated the relative expression of *COMT1* after cold exposure. However, pretreatment with fluridone (50 μm), which can inhibit ABA biosynthesis, completely abolished the pumpkin rootstock-induced upregulation of *COMT1* and accumulation of melatonin under cold stress. Like melatonin, MeJA biosynthesis in watermelon leaves was promoted by pumpkin rootstocks and ABA after cold exposure, as reflected by increased MeJA accumulation and upregulation of *AOC1*, a key gene involved in MeJA synthesis (Figure 5). However, inhibition of ABA biosynthesis by fluridone prevented the pumpkin rootstock-induced MeJA accumulation and *AOC1* expression after cold exposure.

### Involvement of Melatonin and Meja in Rootstock- and ABA-Induced Increase of the Antioxidant Potential and Cold Tolerance

**Figure 6:**
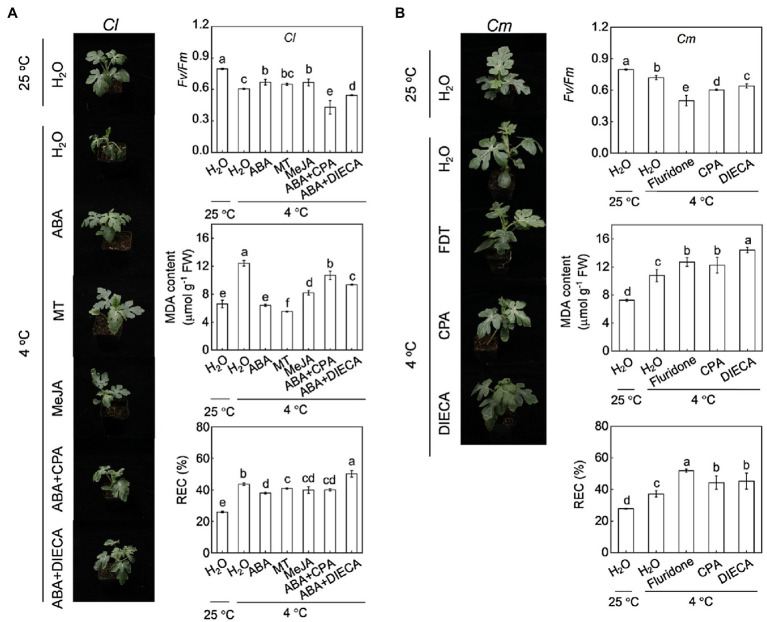
Involvement of melatonin and MeJA in ABA-mediated cold tolerance in grafted watermelon plants. In **(A)**, the leaves of self-grafted (*Cl*) plants were sprayed with ABA (100 μm), melatonin (150 μm), MeJA (200 μm) or H_2_O (Control). Twelve hours later, the plants were exposed to cold stress at 4°C. To inhibit melatonin or MeJA synthesis, the plants were sprayed with p-chlorophenyl alanine (CPA, 100 μm) or diethyldithiocarbamic acid (DIECA, 5 mm), respectively, eight hours prior to ABA application. After cold treatment for 36 h, the phenotypes, photochemical efficiency of PSII (*Fv/Fm*), malondialdehyde (MDA), and relative electric conductivity (REC) were analyzed. In **(B)**, pumpkin-grafted plants (*Cm*) were sprayed with CPA or DIECA 8 hours prior to cold exposure at 4°C. After 36 h of cold treatment, the phenotypes, *Fv/Fm*, MDA, and REC were analyzed. Data are reported as means ± standard deviations (*n* = 3). Different letters indicated significant difference at *p* < 0.05.

Pretreatment with ABA at 100 μm, melatonin at 150 μm, or MeJA at 200 μm enhanced cold tolerance in *Cl* plants, as reflected by the alleviation of plant wilting, increase in *Fv/Fm*, and decrease in MDA and REC (Figure 6A). The MDA content in ABA-, melatonin-, and MeJA-pretreated plants was 35.1, 44.0 and 17.1% lower, respectively, than that in H_2_O-pretreated plants after cold exposure. However, inhibition of ABA biosynthesis by fluridone abolished the pumpkin rootstock-induced alleviation of plant wilting, increase in *Fv/Fm*, and decreases in MDA and REC under cold stress (Figure 6B). Moreover, inhibition of melatonin or MeJA biosynthesis by CPA (100 μm, Supplementary Figure S2) or DIECA (5 mm), respectively, attenuated or abolished the pumpkin rootstock- or ABA-induced increase in *Fv/Fm* and decreases in MDA and REC under cold stress.

**Figure 7:**
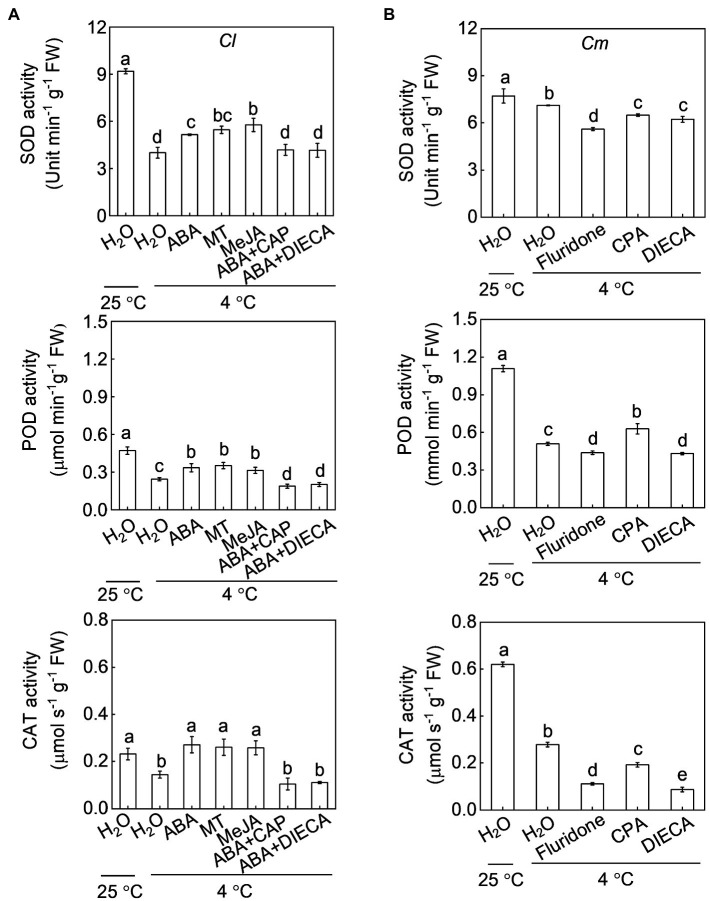
Involvement of melatonin and MeJA in the ABA-induced increase of the antioxidant capacity in grafted watermelon plants under cold stress. The seedlings were treated according to the procedures outlined in Figure 6. After cold treatment for 36 h, the activities of superoxide dismutase (SOD), peroxidase (POD), and catalase (CAT) in (A) self-grafted (*Cl*) and (B) pumpkin-grafted (*Cm*) plants. Data are reported as means ± standard deviations (*n* = 3). Different letters indicated significant difference at *p* < 0.05.

Exogenous application of ABA, melatonin, or MeJA increased the SOD, POD, and CAT activities in *Cl* seedlings under cold stress (Figure 7A). For instance, SOD activity in ABA-, melatonin-, and MeJA-pretreated plants was 28.4, 36.0 and 43.8% higher, respectively, than that in H_2_O-pretreated plants after cold exposure. However, pretreatment with fluridone attenuated or abolished the pumpkin rootstock-induced increase in SOD, POD, and CAT activities under cold stress (Figure 7B). Moreover, pretreatment with CAP or DIECA attenuated or abolished the pumpkin rootstock- or ABA-induced increases in the activities of these enzymes after cold exposure, except that the POD activity was increased by CPA in *Cm* plants.

### Involvement of Melatonin and Meja in Rootstock-Induced ABA Accumulation Under Cold Stress

**Figure 8:**
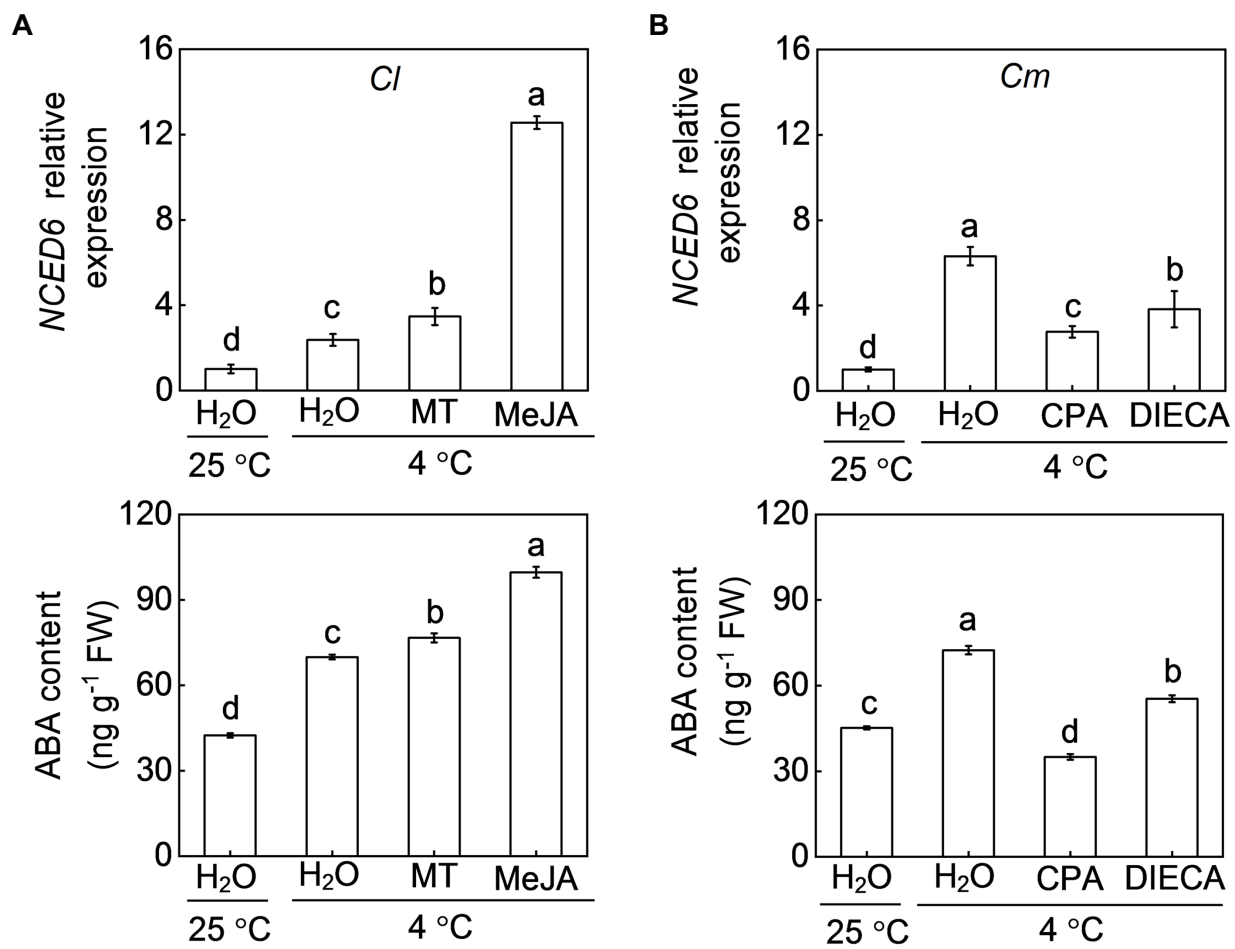
Involvement of melatonin and MeJA in pumpkin rootstock-induced ABA accumulation in leaves under cold exposure. In **(A)**, the leaves of self-grafted (*Cl*) plants were sprayed with melatonin (150 μm), MeJA (200 μm) or H_2_O (Control). Twelve hours later, the plants were exposed to cold stress at 4°C for 12 h. In **(B)**, the leaves of pumpkin-grafted plants (*Cm*) were sprayed with CPA, DIECA, or H_2_O (Control). Eight hours later, the plants were exposed to cold stress at 4°C for 12 h. The relative expression of *9-CIS-EPOXYCAROTENOID DIOXYGENASE 6* (*NCED6*) and ABA levels in leaves of **(A)** self-grafted (*Cl*) and **(B)** pumpkin-grafted (*Cm*) plants were analyzed. Data are reported as means ± standard deviations (*n* = 3). Different letters indicated significant difference at *p* < 0.05.

To investigate whether melatonin or MeJA increase ABA levels in a positive feedback manner, we examined the ABA response to melatonin and MeJA. ABA accumulation in *Cl* leaves after cold exposure was increased by exogenous melatonin (9.7%) and MeJA (42.6%), accompanied by an up-regulation of *NCED6* (Figure 8A). However, CPA or DIECA pretreatment abolished or attenuated the pumpkin rootstock-increased transcript levels of *NCED6* and accumulation of ABA (Figure 8B).

## Discussion

### Melatonin and Meja Are Involved in Rootstock-Alleviated Cold-Induced Oxidative Stress

A common adverse effect of cold stress is the excessive accumulation of ROS by inhibition of the photosynthetic electron transport (Dat et al., 2000). It has been reported that grafting onto tolerant rootstocks can enhance the antioxidant potential and thus alleviate cold-induced oxidative damage in cucumber shoots (Li et al., 2014b). In this study, our results show that grafting onto pumpkin increased the activities of SOD, POD, and CAT and alleviated cold-induced accumulation of O_2_·^−^ and H_2_O_2_ and oxidative damage in watermelon shoots. These findings suggest that pumpkin (*Cucurbita moschata*, a chilling-tolerant species) rootstocks enhance the cold tolerance of watermelon shoots and such role of pumpkin rootstocks is associated with the induction of antioxidant potential (Figures 1, 2).

Melatonin and MeJA are well known to play critical roles in plant responses to multiple environmental stresses (Arnao and Hernández-Ruiz, 2014; Kazan, 2015). Various studies have proven that melatonin is an important antioxidant that can scavenge excess ROS directly or indirectly by inducing an antioxidant system in plants (Zhang and Zhang, 2014; Zhang et al., 2015). Like melatonin, MeJA also enhances stress tolerance by increasing the antioxidant potential and thereby tolerance to oxidative damage (Ali and Baek, 2020). Our recent study has demonstrated that melatonin and MeJA play essential roles and interact with each other in grafting-enhanced watermelon tolerance to cold stress (Li et al., 2021a). During grafted watermelon response to cold stress, melatonin promotes the accumulation of MeJA, which in turn induces melatonin accumulation, forming a forming a self-propagating mutual activation loop that enhances cold tolerance. Consistently, the current results show that pumpkin rootstocks increase the accumulation of melatonin and MeJA in watermelon leaves under cold stress (Figures 4, 5). Pretreatment with melatonin or MeJA increased the antioxidant potential and, consequently, the tolerance to cold-induced oxidative stress in *Cl* plants; however, inhibition of melatonin or MeJA synthesis by pretreatment with CPA or DIECA attenuated rootstock-alleviated oxidative damage under cold stress (Figures 6, 7). Taken together, these data indicate that melatonin and MeJA are involved in the rootstock-induced increase of antioxidant potential and consequent cold tolerance. However, further studies are needed to demonstrate that whether melatonin and MeJA interact with each other to induce antioxidant activity during plant response to cold stress.

### ABA Plays a Vital Role in the Rootstock-Induced Increase of the Antioxidant Potential and Cold Tolerance

Numerous studies have proven that ABA is critical in regulating plant tolerance to various stresses such as cold, heat, drought, and salt (Bulgakov et al., 2019). Root-originated ABA as a long-distance signal plays a crucial role in root-to-shoot communication that regulates plant responses to abiotic stresses (Li et al., 2018). Here, we found that pumpkin rootstocks promote the accumulation of ABA in watermelon leaves after cold exposure (Figure 3). Furthermore, pretreatment with ABA enhanced the antioxidant potential and alleviated cold-induced oxidative stress in *Cl* plants, while pretreatment with fluridone, an ABA synthesis inhibitor, abolished the pumpkin rootstock-induced increase of the antioxidant potential and cold tolerance (Figures 6, 7). These findings indicate that ABA is involved in pumpkin rootstock-induced tolerance to cold and increased antioxidant potential. The ABA content in xylem sap increased in response to cold stress in *Cl* and especially *Cm* plants; however, the rate of ABA exudation reduced (Supplementary Figure S1). This was associated with the decreased sap exudation rate by cold-caused inhibition of root vitality. Notably, the increase of ABA accumulation in *Cm* leaves was accompanied by significant increases in the transcript levels of *NCED6* in leaves, ABA contents in rootstocks, and higher exudation rates of ABA after exposure to cold stress (Figure 3). Therefore, these results suggest that pumpkin rootstock-originated ABA may act as a root-to-shoot signal that partly contributes to the ABA accumulation and consequent cold tolerance of watermelon shoots.

### Interaction Between ABA and Melatonin or Meja in the Rootstock-Induced Increase of the Antioxidant Potential and Cold Tolerance

As two crucial plant growth regulators, the interaction between ABA and melatonin in regulating multiple physiological processes, such as seed germination, stomatal movement, biosynthesis of cuticular waxes, and defense against various abiotic stresses, has been well-documented (Li et al., 2015, 2019, 2020, 2021a,b). However, the mutual effects of ABA and melatonin under stress are still controversial. For instance, exogenous application of melatonin was shown to increase the accumulation of ABA in *Elymus nutans* and cucumber under cold stress (Fu et al., 2017; Zhao et al., 2017). However, melatonin attenuated ABA accumulation by inhibiting the expression of ABA biosynthesis genes under drought and salt stress (Li et al., 2015; Hu et al., 2021). Similarly, melatonin-induced suppression of heat-induced leaf senescence could be associated with inhibiting ABA synthesis and signaling in tomato and perennial ryegrass (Zhang et al., 2017; Jahan et al., 2021). Pretreatment with ABA significantly increased melatonin accumulation under salt stress (Hu et al., 2021), whereas exogenous ABA had almost no effect on melatonin accumulation in *Elymus nutans* response to cold stress (Fu et al., 2017). Therefore, the mutual effects of ABA and melatonin are different in plant responses to different stresses. Here, our results showed that foliar application of ABA and melatonin induces their mutual accumulation, as well as the transcript levels of *COMT1* and *NCED6*, respectively, in the leaves of *Cl* plants after cold exposure (Figures 4A, 8A). Moreover, pretreatment with fluridone and CPA prevented the pumpkin rootstock-induced accumulation of melatonin and ABA, respectively (Figures 4B, 8B). Furthermore, CPA pretreatment abolished the ABA-induced increase of the antioxidant potential and cold tolerance in *Cl* plants (Figures 6A, 7A). These data indicate that ABA and melatonin positively interact with each other to enhance the antioxidant potential and cold tolerance.

ABA and JA function together to regulate plant defense against multiple biotic or abiotic stresses (Moons et al., 1997; Anderson et al., 2004; Adie et al., 2007). ABA is essential for the biosynthesis of JAs, which act downstream of ABA to induce the activation of the *C-REPEAT BINDING FACTOR* pathway and cold tolerance in tomato (Wang et al., 2016). Our results show that, like melatonin, MeJA functions together with ABA to enhance the cold tolerance of grafted watermelon plants. This conclusion is supported by our findings that (1) exogenous ABA and MeJA application increased their mutual accumulation in *Cl* leaves after cold exposure by upregulating the relative expression of *AOC1* and *NCED6*, respectively (Figures 5A, 8A); (2) pretreatment with fluridone or DIECA prevented pumpkin rootstock-induced melatonin or ABA accumulation, respectively (Figures 5B, 8B); and (3) DIECA pretreatment prevented the ABA-induced increase of the antioxidant potential and cold tolerance in *Cl* plants (Figures 6A, 7A).

## Conclusion

In summary, we have revealed in this study that ABA as a potential root-to-shoot signal plays an important role in the rootstock-enhanced cold tolerance of watermelon plants. The rootstock-mediated ABA accumulation in leaves promotes the accumulation of melatonin and MeJA, which in turn induces the accumulation of ABA, forming a positive self-amplifying feedback loop. Melatonin and MeJA increase the antioxidant potential and subsequently alleviate cold-induced oxidative damage. However, it remains elusive how ABA, melatonin, and MeJA interact with each other in grafted plants in response to cold stress.

## Data Availability Statement

The datasets presented in this study can be found in online repositories. The names of the repository/repositories and accession number(s) can be found in the article/Supplementary Material.

## Author Contributions

HL: conceptualization, project administration, and writing-review and editing. YG and HL: data curation, formal analysis, and writing-original draft. XZ and HL: funding acquisition. YG, JY, ZS, JC, and CW: investigation. JY, YZ, JM, and XZ: resources. XZ: supervision. All authors have read and agreed to the published version of the manuscript.

## Funding

This research was funded by the National Key Research and Development Program of China, grant number 2018YFD1000800; the National Natural Science Foundation of China, grant numbers 31801884 and 31972479; the China Agriculture Research System of MOF and MARA, grant number CARS-25; and the Tang Scholar of Northwest A&F University.

## Conflict of Interest

The authors declare that the research was conducted in the absence of any commercial or financial relationships that could be construed as a potential conflict of interest.

## Publisher’s Note

All claims expressed in this article are solely those of the authors and do not necessarily represent those of their affiliated organizations, or those of the publisher, the editors and the reviewers. Any product that may be evaluated in this article, or claim that may be made by its manufacturer, is not guaranteed or endorsed by the publisher.

## Footnotes

^1^ https://www.fao.org/
