# Supplementary material for: Abscisic Acid Mediates Grafting-Induced Cold Tolerance of Watermelon via Interaction With Melatonin and Methyl Jasmonate
Source: Front Plant Sci. 2021 Dec 17;12:785317. doi: 10.3389/fpls.2021.785317 (PMC8719526; doi:10.3389/fpls.2021.785317)
Supplement: Supplementary file 1 [file Data_Sheet_1.docx]

**TABLE S1** Primers used for qRT-PCR analysis

| **Gene ID or accessions** | **Forward sequence (5’-3’)** | **Rreverse sequence (5’-3’)** | **Gene description** |
| --- | --- | --- | --- |
| Cla007792  Cla003512  Cla002942  Cla010664 | CCATGTATGTTGCCATCCAG  AGGTCAAGTTGCAGCAGATCGT  CGGAGATTGGTTGGGCAGAGAA  TCGCCACCAAGGGAGTCATTCA | GGATAGCATGGGGTAGAGCA  AATGGCGGCAGAAGGTTCACAA  CCGTATCTACCGTCGCCGTATA  GCACAGCAGTGGACCTTGAAAC | *β-ACTIN*  *ALLENE OXIDE CYCLASE 1*  *9-CIS-EPOXYCAROTENOID DIOXYGENASE 6 CAFFEIC ACID O-METHYLTRANSFERASE 1* |


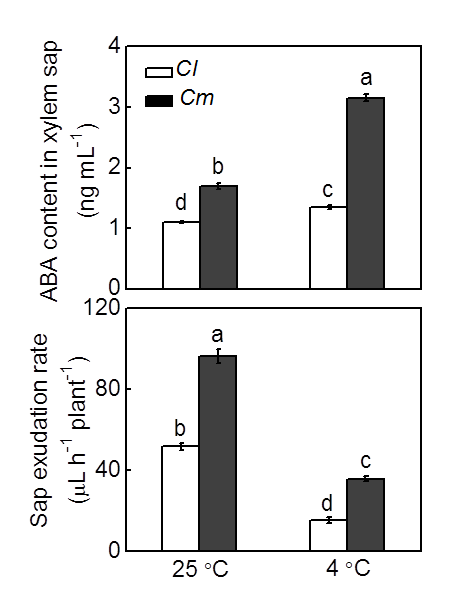


**FIGURE S1** ABA content in xylem sap and sap exudation rate in response to cold in grafted watermelon plants. Self-grafted (*Cl*) and pumpkin-grafted (*Cm*) watermelon plants were exposed to cold stress at 4 °C. Samples of xylem sap were collected at 12 h after cold exposure. Data are reported as means ± standard deviations (n=3). Different letters indicated significant difference at *P* < 0.05.


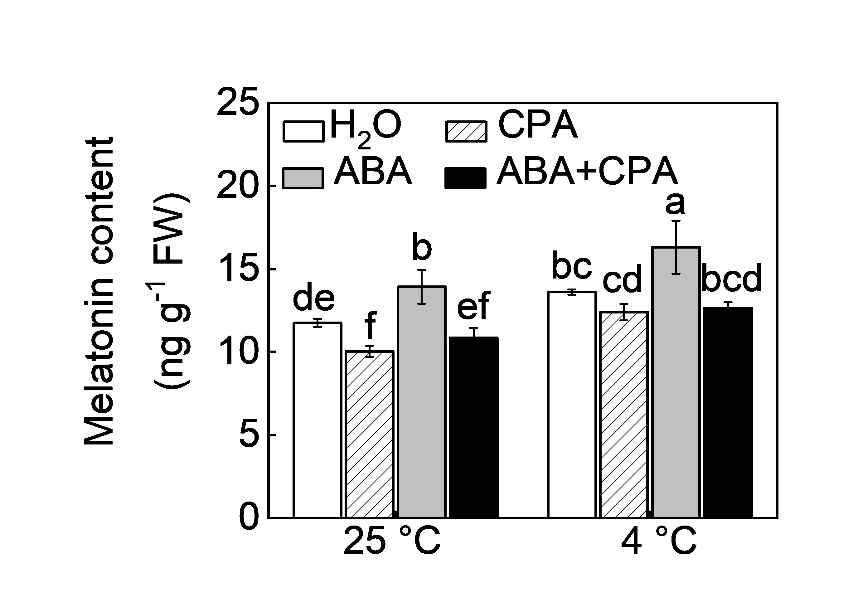


**FIGURE S2** Endogenous melatonin content in response to cold in watermelon plants with ABA and, or p-chlorophenyl alanine (CPA) treatment. Watermelon plants sprayed with 100 μM CPA. After eight hours, the plants were treated with ABA and 12 h later, they were exposed to cold stress at 4 °C. Samples of leaf were collected at 12 h after cold exposure. The experiments were repeated three times, and each experiment contained at least 15 seedlings per treatment. Data are reported as means ± standard deviations (n=3). Different letters indicated significant difference at *P* < 0.05.
